# Chromosomal Inversions between Human and Chimpanzee Lineages Caused by Retrotransposons

**DOI:** 10.1371/journal.pone.0004047

**Published:** 2008-12-29

**Authors:** Jungnam Lee, Kyudong Han, Thomas J. Meyer, Heui-Soo Kim, Mark A. Batzer

**Affiliations:** 1 Department of Biological Sciences, Louisiana State University, Baton Rouge, Louisiana, United States of America; 2 Biological Computation and Visualization Center, Louisiana State University, Baton Rouge, Louisiana, United States of America; 3 PBBRC, Interdisciplinary Research Program of Bioinformatics, College of Natural Sciences, Pusan National University, Busan, Korea; 4 Division of Biological Sciences, College of Natural Sciences, Pusan National University, Busan, Korea; Washington University, United States of America

## Abstract

The long interspersed element-1 (LINE-1 or L1) and *Alu* elements are the most abundant mobile elements comprising 21% and 11% of the human genome, respectively. Since the divergence of human and chimpanzee lineages, these elements have vigorously created chromosomal rearrangements causing genomic difference between humans and chimpanzees by either increasing or decreasing the size of genome. Here, we report an exotic mechanism, retrotransposon recombination-mediated inversion (RRMI), that usually does not alter the amount of genomic material present. Through the comparison of the human and chimpanzee draft genome sequences, we identified 252 inversions whose respective inversion junctions can clearly be characterized. Our results suggest that L1 and *Alu* elements cause chromosomal inversions by either forming a secondary structure or providing a fragile site for double-strand breaks. The detailed analysis of the inversion breakpoints showed that L1 and *Alu* elements are responsible for at least 44% of the 252 inversion loci between human and chimpanzee lineages, including 49 RRMI loci. Among them, three RRMI loci inverted exonic regions in known genes, which implicates this mechanism in generating the genomic and phenotypic differences between human and chimpanzee lineages. This study is the first comprehensive analysis of mobile element bases inversion breakpoints between human and chimpanzee lineages, and highlights their role in primate genome evolution.

## Introduction

Mobile elements make up ∼45% of the human genome [1]. Among them are L1 and *Alu* elements, that have been active since well before the divergence of the human and chimpanzee lineages, and remain active in their host genomes. These two elements mobilize via a “copy and paste” mechanism and integrate into new genomic regions by means of an RNA intermediate [2]. A full-length functional L1 element is about 6 kb in length and able to code for enzymes which are required for L1 retrotransposition, making the L1 an autonomous element [3]. By contrast, the *Alu* element is 300 bp long and does not encode the means of its own retrotransposition, instead borrowing the enzymatic machinery of the L1 elements for its propagation [4], [5], making it a non-autonomous mobile element. Although L1 elements contribute the most to the genome in terms of total size, *Alu* elements are the most successful mobile element family in terms of copy number, reaching a copy number of ∼1.2 million in the human genome [6].

L1 and *Alu* elements have played an important role in shaping their host genomes. They can alter gene expression patterns and cause chromosomal rearrangements through various mechanisms including novel insertion, insertion-mediated deletion, and unequal homologous recombination between elements [7]–[9]. Sequence identity between two retrotransposons of the same type (e.g., *Alu*-*Alu* and L1-L1) can lead to non-allelic homologous recombination between them, that subsequently results in chromosomal rearrangements such as duplications, deletions, translocations, and inversions [9]–[12]. Such recombination can cause species-specific local genomic instability and has been reported as a major source of genomic disorders [13].

Inverted *Alu* and L1 pairs (i.e., two *Alu* elements or two L1 elements inserted in opposite orientations along a chromosome) have caused chromosomal rearrangements in their host genomes through several mechanisms including large inverted duplications, translocations, inversions, and deletions [14]–[16]. Due to their sequence similarity, they have the ability to form a hairpin structure in single-stranded DNA or a cruciform structure in double-stranded DNA [15], [17], [18]. These structures can potentially block progression of the replication fork and cause intra- or inter-molecular template switching of DNA polymerase between the inverted elements [15], [19]. In reality, inverted *Alu* pairs cause a 1000-fold increase in homologous recombination [15]. Here, we report for the first time a genome-wide analysis of retrotransposon recombination-mediated inversion (RRMI), causing genomic and subsequently phenotypic differences between humans and chimpanzees. The previously reported mechanism, *Alu* recombination-mediated deletion (ARMD), alters or interrupts gene function through the deletion of intronic and exonic regions. By contrast, RRMI usually does not cause any change in genome size. Instead, it could alter the structure of genes or transcription of genes by inverting intron or exon sequences and introducing alternative gene splicing sites. Through the comparison of human and chimpanzee draft genome sequences [6], [20], we identified 49 RRMI loci, 28 of which were human-specific inversions and 21 were chimpanzee-specific inversions. Among them, 53% of the RRMI occurred within genic regions. Interestingly, we found that three RRMI events caused alteration of exonic regions in known genes with ten RRMIs that are polymorphic within a species. These findings suggest that recombination between inverted L1 and *Alu* pairs might have generated genomic variation within a species as well as between species.

## Results

### A whole-genome scan for inversion events between human and chimpanzee lineages

To identify potential inversion loci between human and chimpanzee lineages, we computationally compared human with chimpanzee genome reference sequences. We initially obtained a total of 6887 inversion candidates ranging in size from 27 bp to 47.3 Mb and discarded 986 loci whose human chromosomal positions were unknown or random. The remaining 5902 loci were subjected to flanking sequence analysis as described in the materials and methods section. Among them, 3055 loci were categorized as false positives for inversions between the human and chimpanzee genomes. Our computational methodology excluded these loci due to a failure of University of California Santa Cruz (UCSC)'s liftOver utility to find the orthologous positions between the two species. These failures result from species specific-genomic deletions, duplications, or splits, after their removal, a total of 2847 loci were collected as candidate inversion loci.

**Figure 1:**
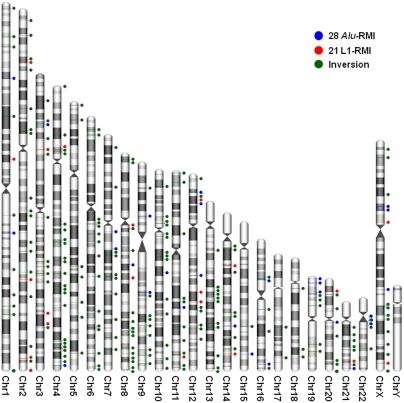
The 252 inversion loci between the human and chimpanzee lineages. Blue and red circles indicate *Alu*-RMI and L1-RMI events, respectively. All inversions except for those caused by RRMI are indicated by green circles. The karyotype images were created using the idiographica webtool [58].

These loci were then subjected to manual inspection. Sequence disagreement between human and chimpanzee genome sequences resulting from the unsequenced regions of the chimpanzee genome and genomic defragmentation [21] significantly reduced our ability to find the inversion breakpoints, especially when the sequence disagreement occurred in the genomic regions where an inversion began or ended. As such, many inversion events may have been eliminated from our data set even though likely to be authentic. Intrachromosomal duplications in which the duplicate is inserted in the reverse direction (inverted duplication) are likely to be a major source of false positives for this analysis. To identify and eliminate them from our data set, we used human inversion sequence as a query for BLAST-like alignment tool (BLAT) against human genome sequence. A false positive showed two highest score hits in the BLAT results, corresponding to the query sequence and the inverted duplication sequence (+ and −, respectively). We removed these false positive inversion loci from our data and finally confirmed 252 inversion events (Figure 1) whose inversion breakpoints are able to be characterized.

### Breakpoint examination for RRMI

To characterize inversion breakpoints, we retrieved human flanking sequence of the 252 inversion loci and used them, combined with the putative inversion sequence, as queries for BLAT searches against the chimpanzee genome sequence (panTro2). The flanking regions, as expected, matched between human and chimpanzee genomes. However, the inverted region stood out clearly, allowing the beginning and end of each inversion, the breakpoints, to be identified.

To identify RRMI events, we examined whether L1s or *Alu* elements spanned the two inversion breakpoints of each inversion event, and whether or not their orientation was opposite to one another. We found 49 RRMI loci (28 *Alu*-RMI and 21 L1-RMI, Table 1, Table S1) out of the 252 inversion events. For example, *Alu*-RMI occurs when two *Alu* elements span the two breakpoints of an inversion and are oriented in opposite directions along the chromosome. Intriguingly, 63 of the remaining 203 inversions were also associated with an L1 or *Alu* element (41 L1- and 22 *Alu*-associated inversions). For these, however, the retrotransposon spanned only one of the two breakpoints, while the other breakpoint was located independently of repetitive elements. One possible explanation for these loci is that microhomology between the retrotransposon and the genomic region where the other inversion breakpoint occurs induced the recombination event responsible for the inversion.

**Table 1:** Summary of retrotransposon recombination-mediated inversion.

Retrotransposon-RMI	Human-specific inversion		Chimpanzee-specific inversion	
	*Alu*-RMI	L1-RMI	*Alu*-RMI	L1-RMI
Total events†	14 (3)	13 (1)	14 (4)	8 (2)
Total inversion size (bp)	27078	185831	11530	25122
Average of inversion size (bp)	1934	14294	769	3140

† The numbers within the parentheses indicate the numbers of RRMI which are accompanied by the deletion of partial inverted sequence.

**Figure 2:**
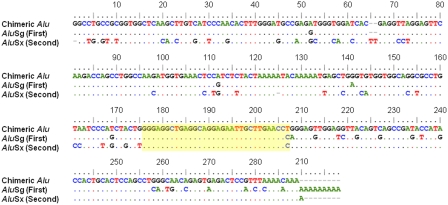
Sequence alignment of one recombined *Alu* element and two prerecombined *Alu* elements involved in an *Alu*-RMI event. The recombined (chimeric) *Alu* element and two prerecombined *Alu* elements that contributed to its formation are showed in order. Identical nucleotides shared among elements are indicated by dots. Otherwise, differences are shown with letters. The recombination breakpoint for this event is located in the yellow box.

When an inversion occurs, the retrotransposons spanning the inversion breakpoints recombine, becoming chimeric elements consisting of the front portion of one element and the back portion of the other. To further characterize the inversion breakpoints of the RRMI loci, we aligned the two ancestral, pre-recombined retrotransposons (e.g., *Alu*Sg and *Alu*Sx) with one of the recombined retrotransposons for each RRMI locus (Figure 2). These alignments allowed more precise determination of where the breakpoints occurred within each element. We counted the frequency of each nucleotide position involved in the windows of the recombination breakpoints on *Alu* and L1 consensus sequences. The frequencies were similar along the length of the consensus sequences, indicating that no recombination hotspot exists in these retrotransposons regarding inversion events between the human and chimpanzee genomes.

### RRMI characterization

As described in the Materials and Methods section, we examined the ancestral state of each RRMI locus using three methodologies. Among the 49 RRMI loci, 27 loci were human-specific inversions whereas 22 loci were chimpanzee-specific inversions. We grouped them into L1-RMI and *Alu*-RMI depending on the type of retrotransposon that spanned the inversion breakpoints. As shown in Table 1, the 49 loci contained 21 L1-RMIs and 28 *Alu*-RMIs.

Inverted repeats frequently cause genomic deletions. We found that genomic deletions were caused even during the inversion process resulting from recombination between inverted repeats. In our data, 12 out of the 49 RRMIs are accompanied by genomic deletions that deleted a portion of the internal sequence and/or the retrotransposons causing the inversion. We extended this examination to the total number of 252 inversion loci identified between humans and chimpanzees and found that ∼30% of the inversion events (75/252) involved genomic deletions of variable sizes ranging from 94 bp to 11,012 bp.

**Figure 3:**
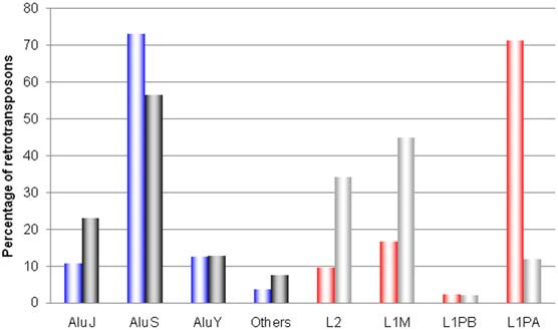
*Alu* and L1 subfamilies involved in RRMI events. The proportion of *Alu* elements involved in *Alu*-RMI events (blue bars) and the proportion of *Alu* elements in each subfamily (black bars) are compared in the left side. The proportion of LINEs involved in L1-RMI events (red bars) and the proportion of L1 elements in each subfamily (gray bars) are compared in the right side.

We further investigated the subfamilies of L1 and *Alu* elements involved in the inversion events. The analysis of *Alu* subfamilies showed that the number of elements from each *Alu* subfamily involved in *Alu*-RMI is proportional to their genome-wide copy number (Figure 3). This result implies that the elements with higher copy numbers are more frequently subjected to recombination than are elements with lower copy numbers. However, more members of the *Alu*Y subfamily are involved in the *Alu*-RMI events than those of the *Alu*J subfamily, even though the *Alu*J subfamily has a higher copy number than the *Alu*Y subfamily in the human and chimpanzee genomes. It is useful to note here that the *Alu*Y subfamily is younger and, therefore, its members tend to have more sequence identity with one another, relative to the *Alu*J subfamily. This suggests that, along with copy number, a high level of sequence identity is also important in the recombination between the two *Alu* elements. This finding is consistent with the patterns described in studies of species-specific ARMD [9], [12]. As shown in Figure 3, the analysis of L1 subfamilies further supports that sequence identity is an important factor affecting the frequency of recombination between these elements. Most LINE members belonging to L2 and L1M subfamilies are older than ∼60 million years while the L1PA subfamilies involved in the inversion events are younger than ∼20 million years [22], [23]. The sequence identities among the members of the L2 or L1M subfamilies are much lower than among the members of the L1PA subfamily because older elements have likely accumulated more substitutions than younger elements. We believe that this high sequence identity has allowed the L1PA subfamily to contribute more frequently to the RRMI events despite their lower copy numbers in the genome relative to other L1 subfamilies.

The RRMI loci range in size from 166 bp to 81,189 bp with an average and a median size of 5364 bp and 1452 bp, respectively. A majority of *Alu*-RMI loci are responsible for the inversions whose sizes are shorter than 1 kb. In contrast, more than half of L1-RMIs are longer than 10 kb. We tested the correlation between the length of elements involved in the inversion event and respective inversion size. This analysis showed a statistically significant positive correlation between the two variables (*r* = 0.578; *p*<0.0001), and suggests that the larger the number of nucleotides capable of base pairing between the two elements the larger the inversion is likely to be. Interestingly, the average size of human-specific inversions is three times longer than that of chimpanzee-specific inversions. This size difference between human and chimpanzee could be explained by a higher efficiency of selection against long inversion in chimpanzees relative to humans. Long inversions are more deleterious to host genome than short inversions are because the chance of recombination between inverted and non-inverted sequences increases as the size of inversion increases. Thus, selection in the host genome acts against long inversions. The efficiency of selection is greater in chimpanzees than in humans because the effective population size of chimpanzees is twice that of humans [24], [25].

### RRMI Polymorphism

Through PCR assays, we verified the integrity of 33 RRMI loci and excluded one chimpanzee-specific inversion locus resulting from sequence assembly error in the chimpanzee genome reference sequence. However, we could not experimentally confirm the remaining loci because they contained a high density of repetitive elements, that inhibit PCR amplification of their respective genomic regions [26]. Additionally, we estimated the polymorphism levels of *Alu*-RMI loci using PCR assay. Nine human-specific *Alu*-RMIs were genotyped in 80 diverse humans (20 individuals from each of four populations, composed of African-American, European, Asian, and South American individuals) and seven chimpanzee-specific *Alu*-RMIs were genotyped in 12 unrelated common chimpanzees. Among them, we identified three human-specific *Alu*-RMI polymorphic loci whose minor allele frequencies were 0.6%, 1.3%, and 43%, respectively. Of the three polymorphic loci, the last has been independently reported through an inversion analysis between the human and chimpanzee genomes [10]. By contrast, only one chimpanzee-specific *Alu*-RMI was found to be polymorphic, and its minor allele frequency was 25%.

Ninety polymorphic inversion loci between the human genome project assembly and the Venter genome sequence were previously reported [27]. Intriguingly, six of the human-specific RRMI loci in our data are found in this data set. We further compared our data with other polymorphic inversion loci in the human population that were previously studied [28], [29]. Among them, three loci were included in our data but these loci overlapped with the six human-specific RRMI loci mentioned above. Thus, it could be stated that at least nine human-specific RRMI loci including the three loci above contribute to genomic variation within the human population. In addition, two of the nine inversion loci show evidence of inverted exonic regions in two known genes, *DOCK3* and *USP40*. *DOCK3* plays an important role in the engulfment of apoptotic cells and in the migration of cells [30], while *USP40* encodes an ubiquitin-specific peptidase 40 that is related to Parkinson disease [31]. A previous study published the mRNA sequence of the human *DOCK3* gene [32]. Levy et al (2007) found this inversion locus to be polymorphic in the human population [23], which means that some human individuals would produce normal mRNA of the *DOCK3* gene. However, we could not rule out that the putative DOCK3 inversion resulted as a consequence of an error in the assembly of human genome sequence (hg18).

### RRMI and the divergence of humans and chimpanzees

Any given inversion locus could be polymorphic within a species but fixed between species. Thus, 27 human-specific RRMIs and 22 chimpanzee-specific RRMIs independently shape their respective genomes, accelerating the genomic divergence between the two species. Our results show that 26 inversions occurred in genic regions while 23 occurred in intergenic regions. Three chimpanzee-specific events are responsible for the inversion of exonic regions in predicted genes, as annotated by the N-SCAN gene prediction tool [33]. In addition, one human-specific inversion involves an exon of the isoform of the *JMJD5* gene (AK310885), which is a putative histone lysine demethylase. Inversions neighboring exons or introns could significantly impact gene function, either by disrupting the gene itself or by generating alternative splice sites or altering gene regulatory networks. Although 23 RRMI events are located in intergenic regions, they could also affect gene expression by locating upstream or on the gene regulatory regions. The effect of RRMI on their host genome is ongoing, leading to continued genomic variation between and within the human and chimpanzee species.

### Environmental characterization of RRMI

**Figure 4:**
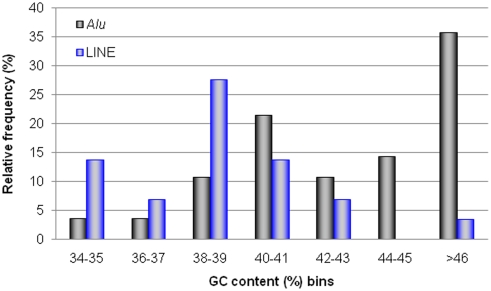
Analysis of GC content in flanking regions of RRMI loci. The vertical axis represents the relative frequency of RRMI loci within each GC bin. Black bars and blue bars indicate *Alu*-RMD and L1-RMD events, respectively.

To estimate the GC content of the genomic regions neighboring the RRMI loci, we extracted 20 kb of flanking sequences (±10 kb in either direction) for each RRMI which does not include the inverted sequence. For this test, we analyzed L1-RMI loci and *Alu*-RMI loci separately because L1s tend to occur in low GC genomic regions while *Alu* insertions preferentially occur in high GC regions [6], [34]. As expected, most of L1-RMI loci were located in GC-poor regions (∼39% GC content, on average) while most of *Alu*-RMIs were found in relatively GC-rich regions (∼44% GC content, on average) (Figure 4). It was recently reported that young *Alu* elements are more ubiquitous in AT rich regions of the human genome [35]. Nonetheless, our results showed that seven out of eight inversion events caused by the *Alu*Y subfamily occurred in genomic regions with GC contents higher than 41%, the genome-wide average [6].

We estimated the gene density of the genomic regions flanking RRMI loci by counting the number of known or predicted genes in the 4 Mb of the flanking sequences (±2 Mb in either direction). The gene density of the regions neighboring *Alu*-RMI loci is estimated to be one gene per 60 kb, on average. This estimate of the gene density is congruent with the gene density of the flanking regions of ARMD loci in the human and chimpanzee genomes [9], [12]. This is an expected result because *Alu*-RMI and ARMD events both result from the same mechanism, recombination between *Alu* elements. In contrast, the gene density of the regions neighboring L1-RMI loci is estimated to be one gene per 98 kb which is similar to the global average gene density in the human genome (one gene per 94 kb). Despite the fact that L1-RMI events were located, on average, in less gene-dense regions of the genome than their *Alu*-RMI counterparts, we found that five out of the six RRMI events that caused the inversion of exonic regions within known and predicted genes were L1-RMI events.

GC content is positively correlated with gene density and the local chromosomal recombination rate [6], [36], [37]. Our results based on GC content, gene density, and frequencies of *Alu*-RMI and L1-RMI are largely congruent. However, we found one interesting locus that resulted from the recombination between two L2 elements. L2 is an ancestor of L1 and, therefore, presumably inserted to host genome several hundred millions ago [23]. As the time an element resides in a specific genomic locus increases, more nucleotide substitutions accumulate in the elements. This age-related degradation significantly reduces the nucleotide identity between members of L2 subfamily. We investigated this locus in detail and discovered that its flanking sequence has a GC content of 59%. High sequence identity between L1 elements efficiently promotes recombination between them regardless of the GC contents of the chromosomal regions in which they reside. However, in cases where the sequence identity between the elements is relatively low, high GC content may promote recombination between L1 elements because GC content, as stated above, positively correlates with the local recombination rate.

## Discussion

### Identification of inversions between humans and chimpanzees

An inversion results from two breaks on a single chromosome followed by a reversal of the orientation of the chromosomal segment between the breaks [38]. This mechanism is unlikely to result in insertion and deletion events, and usually does not result in a change in genome size, which makes the identification of these events more difficult. This characteristic, combined with imperfect genome assemblies, makes the estimation of a precise number of inversions between these lineages difficult. As an example, a previous comparative study identified 1576 putative inversions [10], but this data set included a large fraction of false positives, likely resulting from the use of the lower quality early sequence assembly of the chimpanzee genome (panTro1) for comparison with the human genome. Our study uses a comparison between the highest quality genome assemblies currently available, and identified a total of 323 inversion loci between the human and chimpanzee lineages, regardless of whether they have precise inversion breakpoints. However, this number is likely to be an underestimate because of the method we used to validate candidate inversion events (see Materials and Methods). Large inversions are particularly likely to be eliminated from our data because they are more frequently subjected to species-specific chromosomal rearrangements. This leads to sequence disagreement between inverted and non-inverted sequences, making identification of the original inversion difficult.

Two previous studies identified inversion events in the human population. They found 56 and 224 inversions by using fosmid paired-end sequences and a combination of a clone-based method and fluorescence *in situ* hybridization, respectively [28], [29]. Given the assumption that the frequency of inversion is constant through time, there should be at least several thousands of inversion events between the human and chimpanzee genomes. Thus, finer reference sequences for both genomes and more sensitive identification techniques are required to better estimate the number of inversion events between the two species.

### Impact of inversions on the genomic variation between humans and chimpanzees

Chromosomal rearrangements are thought to be important in the speciation events separating the human from its nearest extant relative, the chimpanzee [39], [40]. Among them, chromosomal inversions, including nine pericentric inversions, have been considered major drivers in the speciation process [39], [41], [42]. These chromosomal inversions not only contributed to the speciation events in the human and chimpanzee lineages, but also contribute to their current genomic variation as described below.

It has been suggested that inversion events suppress recombination in surrounding regions because recombination between inverted and non-inverted sequences is less likely to occur [38], [40]. We examined the local recombination rates of the chromosomal regions where the human-specific RRMIs reside, by using the UCSC genome browser. We found that a majority of human-specific RRMIs reside within chromosomal regions with low local recombination rates. Thus, this result supports that inversion events reduce the recombination rates of their respective chromosomal regions.

Half of the RRMI events identified involve exonic or intronic regions. The inversion of an exonic region could cause non-functionalization of related genes and the inversion of intronic regions could result in alternative splicing patterns, affecting the level of gene expression. In addition, three inversions existing in intronic regions are polymorphic within a species, a result which we experimentally confirmed. RRMIs have therefore contributed to the genomic variation between and within the human and chimpanzee lineages, and some of these genomic variations could have led to phenotypic variation between the two species.

### Role of *Alu* and L1 in causing inversion events

It has been speculated that mobile elements are one of the factors contributing to chromosomal inversions between the human and chimpanzee lineages. Here, we comprehensively analyzed retrotransposon-mediated inversion between the two species. Among the 252 inversion loci identified, 49 inversions were found to have been caused by inverted L1 or *Alu* pairs. In addition, 41 and 22 inversions were also associated with L1 and *Alu* elements, respectively. For these loci, an L1 or *Alu* spanned only one of the two inversion breakpoints. Interestingly, one out of the 41 L1-associated inversions was caused by two L1 elements, but they were oriented in the same direction, contrary to the expected pattern for RRMI. One possible explanation for this locus is that double-strand breaks (DSBs) occurred within the two L1s, after which the internal sequence was reversely oriented and the breaks repaired. This suggests that L1 and *Alu* elements could serve as fragile sites that tend to result in chromosomal breaks or gaps leading to inversions [43]. In total, L1 and *Alu* elements are shown to be responsible for approximately ∼44% (112/252 events) of the total inversions between the human and chimpanzee lineages.

Along with retrotransposons, segmental duplications are considered to be major factors contributing to chromosomal inversion [43]–[47]. Sequence identity between the inverted segmental duplications is high enough to cause non-allelic homologous recombination and thus facilitates chromosomal inversion [43]. In addition, the comparison of human and mouse genome sequences showed that the segmental duplications are highly related to chromosomal breakpoints in the inversion areas [48]. This finding strongly supports the relationship between segmental duplications and chromosomal inversions because a chromosomal break is a necessary step in generating an inversion. Interestingly, *Alu* elements have been suspected as prime candidates to mediate the formation of segmental duplications. This is supported by the fact that the formation of most segmental duplications coincides with the timing of a burst in *Alu* amplification beginning ∼35 million years ago [49], [50]. Taken together, *Alu* elements and L1s have a high potential to have mediated the chromosomal inversions observed between the human and chimpanzee lineages.

### Inverted repeats and genomic instability

L1 and *Alu* elements are the most abundant mobile elements in the human and chimpanzee genomes [6], [20] and thus L1 and *Alu* pairs that are inverted in their orientation relative to one another are common throughout the genomes. These inverted repeats have been considered as hotspots in causing chromosomal rearrangements. Base pairing between inverted L1 or *Alu* pairs can form single-stranded hairpin structures, the formation of which is spontaneous due to the low free energy of the hairpin structure (e.g. the most probable hairpin formation has a ΔG of −12.4 kcal/mol) [51]. This hairpin structure places the chromosomal regions adjacent to the elements involved into close physical proximity, increasing the likelihood of DSB, recombination, and replication slippage on the regions flanking the stem loop structure. Any DSB could be repaired by non-allelic homologous recombination or non-homologous end joining, resulting in genomic inversions or deletions. In case where recombination between the inverted repeats results in an inversion of the internal sequence, the recombination rate between the inverted repeats is positively related to the size of the repeats but negatively related to the distance between the repeats [52]. Thus, inverted L1 pairs are able to induce the inversion of longer genomic sequences than inverted *Alu* pairs, as shown in our results (Table 1). Inverted L1 and *Alu* pairs not only facilitate recombination between themselves, but also increase local recombination rate on their respective chromosomal regions. One previous study reported that inverted repeats increased intrachromosomal and interchromosomal recombinations on their flanking regions 2400-fold and 17000-fold, respectively [53]. In addition, the inverted repeats cause interchromosomal effects by acting as hotspots for mitotic interchromosomal recombination [53].

During DNA replication, single-stranded DNA can form a secondary structure by allowing base pairing between inverted L1 and *Alu* pairs, which may predispose DNA polymerase to slip on the replication template, leading to the deletion of some genomic regions. The genomic deletion caused by inverted repeats have been well studied in various organisms, including bacteria, yeast, and human [52]–[54]. Although *Alu* elements are evenly distributed throughout the genome in terms of their orientation, when *Alu* pairs whose internal sequence is shorter than 650 bp were counted, two-thirds of the total number of *Alu* pairs belong to non-inverted *Alu* pairs in the human genome. However, as the length of the internal sequence increases, the proportions of the non-inverted and inverted *Alu* pairs become balanced [55]. These findings suggest that inverted repeats located close to one another are more unstable in host genomes.

In conclusion, our study supports that inverted repeats could have played an important role in genome variation between and within the human and chimpanzee lineages. Although the number of inverted L1 and *Alu* pairs is similar between human and chimpanzee, they have shaped different chromosomal regions in independent ways, accelerating genomic variation and subsequent phenotypic variation between the two lineages. In this study, we conducted a genome-wide analysis of RRMI between the human and chimpanzee lineages. However, more detailed studies about other chromosomal rearrangements that may be caused by inverted repeats are required to understand the full extent of their role in chromosomal evolution and speciation.

## Materials and Methods

### Computational data mining and manual inspection for RRMI loci

For the comparison of human and chimpanzee genome reference sequences, we utilized the March 2006 freeze of the human (*Homo sapiens*) genome and the March 2006 freeze of the chimpanzee (*Pan troglodytes*) genome from the UCSC. To identify potential RRMI events between the two genomes, we first found all putative inversion loci between them, based on UCSC Table Browser utility, comparing human to chimpanzee genome reference sequences (http://genome.brc.mcw.edu/cgi-bin/hgTables?org=Human&db=hg18&hgsid=2066727&hgta_doMainPage=1). After obtaining the human and chimpanzee genomic positions for each inversion locus, we extracted 15 kb of flanking sequence in either direction of the human genomic position. By using UCSC's liftOver utility (http://genome.brc.mcw.edu/cgi-bin/hgLiftOver), we obtained the orthologous positions within the chimpanzee genome reference sequence that corresponded to the human flanking sequences. If liftOver failed to return an orthologous position in the chimpanzee genome, the locus was discarded. The remaining inversion loci were subjected to manual inspection. We extracted the inverted human sequence and 1 kb of flanking sequence in either direction of the inversion. Next, the human sequence was used as a query to search against the chimpanzee genome sequence using UCSC's BLAT. For each hit in the BLAT search, we retrieved the human and chimpanzee sequences and annotated repeat elements existing in the sequences utilizing RepeatMasker (http://www.repeatmasker.org/cgi-bin/WEBRepeatMasker) analysis. In the case of authentic inversions between the human and chimpanzee genomes, the RepeatMasker output would show that the order and direction of repetitive elements in the human loci were reversed relative to their chimpanzee counterparts.

### PCR amplification and DNA sequencing

RRMI loci were verified by PCR assay with four different DNA templates including human, chimpanzee, gorilla, and orangutan. Cell lines used to isolate the DNA samples were as follows: *Homo sapiens* (HeLa; ATCC CCL-2), *Pan troglodytes* (common chimpanzee Clint: AG06939B), *Gorilla gorilla* (western lowland gorilla: AG05251), and *Pongo pygmaeus* (orangutan; AG05252A).

Oligonucleotide primers for each RRMI locus were designed using Primer3 software (http://www-genome.wi.mit.edu/cgi-bin/primer/primer3_www.cgi) and then computationally tested utilizing both the Oligonucleotide Properties Calculator [56] and UCSC's In-Silico PCR (http://genome.ucsc.edu/cgi-bin/hgPcr?command=start). The primers were then used to amplify RRMI loci (Table S2). Each PCR amplification was performed in 25 µl reactions with 10–50 ng DNA, 200 nM of each oligonucleotide primer, 200 µM dNTPs in 50 mM KCl, 1.5 mM MgCl_2_, 10 mM Tris-HCl (pH 8.4), and 2.5 units Taq DNA polymerase. The conditions for the PCR were an initial denaturation step of 5 min at 95°C, followed by 32 cycles of PCR at 15 sec of denaturation at 95°C, 30 sec at the annealing temperature, and 1 min of extension at 72°C, followed by a final extension step of 10 min at 72°C. The PCR products were loaded on 1–2% agarose gels, depending on the product sizes, stained with ethidium bromide, and visualized using UV fluorescence (Bio-Rad, Hercules, CA). In cases where the expected size of the PCR product was greater than 1.2 kb, iTaq (Bio-Rad, Hercules, CA), Ex Taq polymerase (TaKaRa, Otsu, Shiga, Japan) or KOD Hifi DNA polymerase (Novagen, Madison, WI) were used following the manufacturer's instructions.

If needed, individual PCR products were purified from the agarose gels using the Wizard gel purification kit (Promega, Madison, WI) and cloned into vectors using TOPO-TA Cloning kit (Invitrogen, Carlsbad, CA) according to the manufacturer's instructions. For each sample, three colonies were randomly selected and subject to colony PCR. The sequencing of the colony PCR products was performed using dideoxy chain-termination sequencing on an Applied Biosystems ABI3130*XL* Genetic Analyzer (Applied Biosystems, Foster City, CA). Raw sequence data were analyzed using DNASTAR's Seqman program in the Lasergene version 5.0 software package (http://www.dnastar.com).

### Identification of ancestral state for RRMI

**Figure 5:**
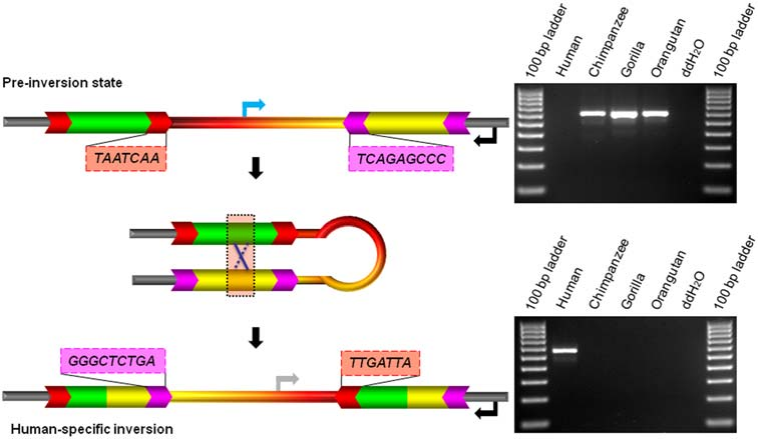
RRMI between human and chimpanzee lineages. The mechanism underlying RRMI is shown at the left. In the illustration of the ancestral state, the two retrotransposons have intact TSDs whose sequence is listed in the colored boxes. The shape “X” indicates recombination between the retrotransposons. In the illustration of the human-specific inversion, both retrotransposons are chimeric, and no longer have matching TSDs. For both illustrations, two arrows indicate the positions where each oligonucleotide primer anneals to for PCR amplification. Agarose-gel chromatographs of PCR products are shown on the right. The upper gel picture displays the ancestral state of the RRMI, while the lower gel picture displays the human-specific inversion. The DNA templates used in each PCR reaction are shown on top of the gel pictures.

To identify the ancestral (i.e., pre-inversion) state of each RRMI locus, we combined three methods: target-site duplication (TSD) analysis, BLAT search, and PCR assay. L1 and *Alu* elements are accompanied on both sides by short direct repeats termed TSDs, which range in size from 7 to 20 bp and are nearly identical to one another [57]. Each element tends to have unique TSDs and rarely share TSD sequences with other elements. Given this, we scrutinized the TSDs of the L1 and *Alu* elements that spanned each inversion breakpoint (Figure 5). If an RRMI event had occurred, the breakpoint-spanning elements would become chimeric, and the TSDs for these elements would no longer match one another. The determination of the ancestral state of each locus could therefore be made based upon the presence of matching TSDs.

Identification of the ancestral state using BLAT searches involved the use of orangutan and rhesus macaque as out groups. We used the human inverted sequences as queries for BLAT searches against four genome assemblies: the human (hg18), chimpanzee (panTro2), orangutan (ponAbe2), and rhesus macaque (rheMac2). Human-specific inversions were characterized by a pattern in which all genomes except the human showed similar orientation patterns in the graphical results window provided by BLAT. In contrast, cases of chimpanzee-specific inversions produced patterns in which only the chimpanzee genome showed different graphical patterns from the others.

For those RRMI loci whose ancestral state was still ambiguous, despite both TSD and BLAT analyses, we experimentally confirmed the ancestral state using PCR assays. We designed one oligonucleotide primer from the flanking sequence of the inversion and the other from the internal sequence between two repeats. To decide the ancestral state of the RRMI, we then compared PCR products from human, chimpanzee, gorilla, and orangutan (Figure 5).

### Analysis of RRMI franking sequences

To estimate the gene density of genomic regions neighboring the RRMI loci, we counted the number of genes within the 4 Mb of sequence flanking the 5′ and 3′ ends of each RRMI locus, using the National Center for Biotechnology Information Map Viewer utility, run on Build 36.3 of the *Homo sapiens* genome and Build 2.1 of the *Pan troglodytes* genome (http://www.ncbi.nlm.nih.gov/mapview). For GC content analysis, 10 kb of flanking sequence in either direction of each RRMI locus was collected. The GC content of the combined 20 kb of flanking sequences was then calculated using the Mobyle geecee utility (http://mobyle.pasteur.fr/cgi-bin/MobylePortal/portal.py?form=geecee).

## Supporting Information

Table S1 Genomic positions of RRMI loci between human and chimpanzee lineages
(0.03 MB XLS)

Table S2 Primer information for RRMI loci
(0.03 MB XLS)
